# Clinical and molecular characterization of an 18‐month‐old infant with autosomal recessive cutis laxa type 1C due to a novel *LTBP4* pathogenic variant, and literature review

**DOI:** 10.1002/mgg3.735

**Published:** 2019-05-21

**Authors:** Marco Ritelli, Francisco Cammarata‐Scalisi, Valeria Cinquina, Marina Colombi

**Affiliations:** ^1^ Division of Biology and Genetics, Department of Molecular and Translational Medicine University of Brescia Brescia Italy; ^2^ Unit of Medical Genetics, Department of Pediatrics, Faculty of Medicine University of the Andes Mérida Venezuela

**Keywords:** ARCL1C, autosomal recessive cutis laxa type 1C, latent transforming growth factor‐beta binding protein 4, *LTBP4*

## Abstract

**Background:**

Cutis laxa (CL) is a group of rare connective tissue disorders mainly characterized by wrinkled, redundant, inelastic, and sagging skin. Besides skin anomalies, in most CL forms multiple organs are involved, leading to severe multisystem disorders involving skeletal, cardiovascular, pulmonary, and central nervous systems. CL might be challenging to diagnose because of its different inheritance patterns, extensive phenotypic variability, and genetic heterogeneity. Herein, we report the clinical and molecular characterization of an 18‐month‐old infant with signs suggestive of recessive cutis laxa type 1C (ARCL1C), although with a relatively mild presentation.

**Methods:**

To confirm the clinical suspicion, mutational screening of all the exons and intron‐flanking regions of the latent transforming growth factor‐beta binding protein 4 gene (*LTBP4*) was performed by Sanger sequencing on an ABI3130XL Genetic Analyzer.

**Results:**

Apart from the presence of the dermatological hallmark, the reported patient did not show pulmonary emphysema, which is the most common and discriminative finding of ARCL1C together with gastrointestinal and urinary involvement. Indeed, pulmonary involvement only included episodes of respiratory distress and diaphragmatic eventration; intestinal dilation and tortuosity and hydronephrosis were also present. Molecular analysis disclosed the novel homozygous c.1450del (p.Arg484Glyfs*290) pathogenic variant in exon 12 of *LTBP4*, thus leading to the diagnosis of ARCL1C.

**Conclusion:**

Our findings expand both the knowledge of the clinical phenotype and the allelic repertoire of ARCL1C. The comparison of the patient's features with those of the other patients reported up to now offers future perspectives for clinical research in this field.

## 1 INTRODUCTION

Cutis laxa (CL) refers to a heterogeneous group of rare connective tissue disorders characterized by wrinkled, redundant, inelastic, and sagging skin. Both hereditary and acquired forms exist. The latter appear secondary to infections, administration of medications or as a paraneoplasm, whereas inherited CL is caused by structural abnormalities of the extracellular matrix (ECM). Different inborn metabolic errors have also been found to be associated with CL (Berk, Bentley, Bayliss, Lind, & Urban, 2012; Gardeitchik & Morava, 2013; Mohamed, Voet, Gardeitchik, & Morava, 2014). The inheritance can be autosomal dominant, autosomal recessive and X‐linked recessive, and 13 causative genes have been identified yet (Mohamed et al., 2014; Van Damme et al., 2017).

Among the different hereditary forms (incidence 1/400,000), the autosomal recessive form (ARCL) is the most prevalent and heterogeneous type (Gardeitchik & Morava, 2013; Morava, Guillard, Lefeber, & Wevers, 2009). Indeed, ARCL is divided into two major types and several subtypes based on the variable phenotypes and underlying defects in different genes. Given the phenotypic overlap among the different forms, proper differential diagnosis might be challenging as well as predicting the causative gene according to the clinical picture of patients, mainly due to the lack of detailed comparative phenotype data (Berk et al., 2012; Gardeitchik & Morava, 2013; Morava et al., 2009). ARCL1 type 1 is subdivided in ARCL1A (MIM #219100), which is caused by pathogenic variants in the fibulin‐5 gene (*FBLN5*, MIM *604,580) (Elahi et al., 2006; Hu et al., 2006; Loeys et al., 2002; Tekedereli et al., 2019), ARCL1B (MIM #614437) caused by mutations in the EGF‐containing fibulin‐like extracellular matrix protein 2 gene or fibulin‐4 gene (*EFEMP2* or *FBLN4*; MIM *604,633) (Dasouki et al., 2007; Hucthagowder et al., 2006; Letard et al., 2018), and ARCL1C (MIM #613177), a.k.a. Urban‐Rifkin‐Davis syndrome, which is due to biallelic variants in the latent transforming growth factor‐beta binding protein 4 gene (*LTBP4*, MIM *604,710) (Callewaert et al., 2013; Su et al., 2015; Urban et al., 2009). ARCL type 2 is separated into ARCL2A (MIM #219200), caused by mutations in the gene encoding for the H^+^ transporting α2 subunit of the vesicular ATPase complex (*ATP6V0A2,* MIM *611,716) (Fischer et al., 2012; Hucthagowder et al., 2009; Kornak et al., 2008; Ritelli et al., 2014), ARCL2B (MIM #612940) that results from mutations in the pyrroline‐5‐carboxylate reductase 1 gene (*PYCR1*; MIM *179,035) (Dimopoulou et al., 2013; Reversade et al., 2009; Ritelli et al., 2017), and ARCL2C (MIM #612940) and ARCL2D (MIM #617403) that are due to biallelic variants in the ATPase H^+^ transporting V1 subunits E1 (*ATP6V1E1*; MIM *108,746) and A (*ATP6V1A*, MIM *607,027), respectively (Alazami et al., 2016; Van Damme et al., 2017). De Barsy syndrome (DBS), previously known as ARCL3A (ARCL3A; MIM #219150), forms a phenotypic continuum with ARCL2 and patients with DBS have been characterized for mutations in the aldehyde dehydrogenase 18 gene (*ALDH18A1*, MIM *138,250) (Guernsey et al., 2009; Skidmore et al., 2011) as well as in A*TP6V0A2* and *PYCR1* (Leao‐Teles, Quelhas, Vilarinho, & Jaeken, 2010; Zampatti et al., 2012). Additional recessive conditions with CL‐like phenotypes include “macrocephaly‐alopecia‐cutis laxa‐scoliosis syndrome” (MACS, MIM #613075), which is caused by mutations in the RAS and RAB interactor 2 gene (*RIN2*; MIM *610,222) (Aslanger et al., 2014; Basel‐Vanagaite et al., 2009), and geroderma osteodysplasticum (GO; MIM #231070) caused by mutations in the Golgi, RAB6‐interacting gene (*GORAB*, MIM *607,983) (Hennies et al., 2008).

Besides skin anomalies, in most ARCL forms multiple organs are involved, leading to severe multisystem disorders involving skeletal, cardiovascular, pulmonary, and central nervous systems (Gardeitchik & Morava, 2013; Mohamed et al., 2014). In particular, ARCL1 patients fall within the severe end of the phenotypic spectrum (Callewaert & Urban, 2016; Loeys, Paepe, & Urban, 2001; Van Maldergem & Loeys, 2009). Inguinal/umbilical hernias, vesicourinary and gastroesophageal reflux and/or diverticula are present in all patients and bladder/intestinal diverticula and/or pyloric stenosis together with CL are considered pathognomonic (Callewaert & Urban, 2016; Loeys et al., 2001; Mohamed et al., 2014; Van Maldergem & Loeys, 2009). Likewise, severe pulmonary emphysema is the most common and discriminative finding, since it has been described in all ARCL1 subtypes. Cardiac involvement might be variable and includes peripheral pulmonary artery or supravalvular aortic stenoses. The disease course depends on the cardiovascular and pulmonary involvement. Lung emphysema, recurrent pulmonary infections and cardiac failure determine the long‐term survival and most children die in early childhood (Callewaert & Urban, 2016; Loeys et al., 2001; Van Maldergem & Loeys, 2009). Differential diagnosis between different ARCL1 subtypes relies on the presence/absence of gastrointestinal and urinary involvement (less gastrointestinal involvement in *EFEMP2* compared to *FBLN5*, urinary diverticula in *EFEMP2*, and severe involvement of both systems in *LTBP4*). *EFEMP2*‐related CL patients have severe arterial tortuosity with predisposition for aneurysms/dissections, which is rare in the other subtypes (Callewaert & Urban, 2016; Loeys et al., 2001; Mohamed et al., 2014; Van Maldergem & Loeys, 2009).

Herein, we report on an 18‐month‐old Venezuelan female with signs suggestive of ARCL1 and compare the patient's features with those of the other individuals with *LTPB4*‐related CL reported up to now.

## 2 PATIENT AND METHODS

### 2.1 Ethical compliance

This study follows the Helsinki Declaration's principles and was carried out from routine diagnostic activity; formal ethics review was therefore not requested. The patient's parents provided written informed consent for and publication of clinical data and photographs. The patient was evaluated at the Unit of Medical Genetics (Department of Pediatrics) of the University Hospital of Mérida in Venezuela. Genetic testing was performed at the Division of Biology and Genetics (Department of Molecular and Translational Medicine) of the University of Brescia in Italy.

### 2.2 Molecular analysis

After informed consent was obtained from the patient's parents, molecular characterization was performed on genomic DNA purified from peripheral blood leukocytes using standard procedures. All of the exons and intron‐flanking regions of the *LTBP4* gene (reference sequences: NG_021201.1 NM_003573.2, NP_003564.2) were PCR amplified by using optimized genomic primers (available upon request) that were analyzed for the absence of known variants using the GnomAD database (https://gnomad.broadinstitute.org/). PCR products were purified with ExoSAP‐IT (USB Corporation) followed by bidirectional sequencing with the BigDye Terminator v1.1 Cycle Sequencing kit on an ABI3130XL Genetic Analyzer (Applied Biosystems). The sequences were analyzed with the Sequencher 5.0 software (www.genecodes.com) and variants were annotated according to the Human Genome Variation Society (HGVS) nomenclature by using the Alamut Visual software version 2.11 (www.interactive-biosoftware.com). The novel pathogenetic *LTBP4* variant identified in the patient was submitted to the Leiden Open Variation Database (LOVD).

## 3 RESULTS

**Figure 1:**
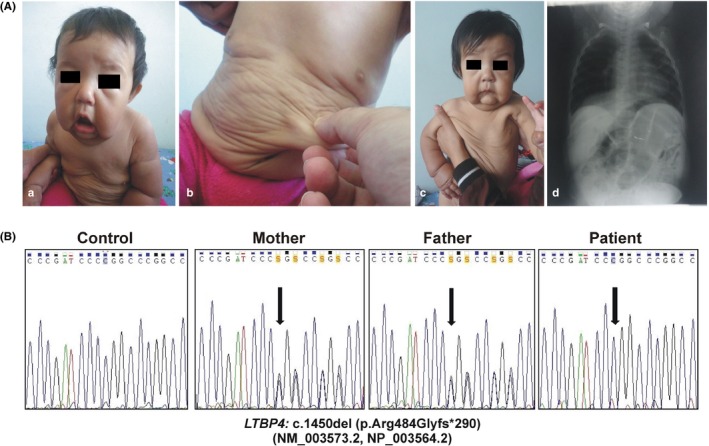
(A) Clinical appearance of the patient. At 9 months of age (a, b), a diagnosis of CL was given for the presence of loose, wrinkled, sagging, and redundant skin, and several craniofacial features. On examination at 13 months of age (c), normocephaly (between 25th and 50th percentile), dysmorphisms, that is, narrow forehead, down slanting palpebral fissures, periorbital fullness, epicanthus, hypertelorism, long philtrum, fat midface, depressed nasal bridge, anteverted nares, micro‐retrognathia, and short neck were observed. Cutis laxa was evident on cheeks (with a prematurely aged appearance), neck, axillae, arms, abdomen, glutei, and limbs. Thorax and abdomen radiography, performed at 10 months of age (d), showed discreetly prominent aortic arch with mild tortuosity, diaphragmatic eventration, normal pulmonary parenchyma with atelectasis in the left lung, and elongated gastrointestinal tract with dilatation and tortuosity. (B) Molecular analysis. Sequence chromatograms showing the position of the c.1450del (p.Arg484Glyfs*290) variant (arrows) identified in the patient in homozygosity in exon 12 of the *LTBP4* gene. Both healthy parents were heterozygous carriers. Mutation is annotated according to HGVS nomenclature (http://www.hgvs.org/mutnomen; NM_003573.2, NP_003564.2)

The patient was born at 39 weeks of gestation from consanguineous (cousins) unaffected Venezuelan parents via an uneventful, spontaneous vaginal delivery. At birth, her weight was 3.6 kg (1.2 *SD*) and length 50 cm (0.6 *SD*). Clinical history was remarkable for perinatal respiratory distress and neonatal hypotonia. Delayed anterior fontanel closure and postnatal growth retardation were also reported. At 9 months, a clinical diagnosis of CL was given for the presence of the dermatological hallmark, that is, loose, wrinkled, sagging, and redundant skin (Figure 1A a,b). At 10 months, heart ultrasound revealed a small interatrial septal defect without hemodynamic repercussion and renal ultrasound right pyelocalicial ectasia and hydronephrosis. On examination at 13 months, several craniofacial features were observed, that is, narrow forehead, down slanting palpebral fissures, periorbital fullness, epicanthus, hypertelorism, long philtrum, fat midface, depressed nasal bridge, anteverted nares, posteriorly rotated ears, micro‐retrognathia, and short neck. The skin was inelastic, sagging, and redundant on cheeks (with a prematurely aged appearance), neck, axillae, arms, abdomen, glutei, and limbs (Figure 1A c). An umbilical hernia was present. Delayed psychomotor development, hypotonia, and hypermobility of small joints were also observed. Thorax/abdomen radiography showed discreetly prominent aortic arch with mild tortuosity, diaphragmatic eventration, normal pulmonary parenchyma with atelectasis in the left lung, and elongated gastrointestinal tract with dilatation and tortuosity (Figure 1A d). At 18 months, she was hospitalized for pneumonia with significant respiratory distress, successfully treated with antibiotics and oxygen supplementation.

Considering the patient's cutaneous and craniofacial features, the presence of respiratory distress, diaphragmatic eventration, and hydronephrosis, and the absence of major vascular, skeletal and central nervous systems' involvement, ARCL1C was supposed. Sanger sequencing of the *LTBP4* gene (NM_003573.2, NP_003564.2) confirmed the clinical suspicion disclosing the homozygous c.1450del variant leading to frameshift and formation of a premature termination codon (PTC) (p.Arg484Glyfs*290). Both parents were heterozygous carriers (Figure 1b). The variant (hg19/GRCh37:g.41114443del) was not found in population and disease databases including gnomAD (https://gnomad.broadinstitute.org/), Bravo (https://bravo.sph.umich.edu/freeze5/hg38/), ClinVar (https://www.ncbi.nlm.nih.gov/clinvar/), and LOVD (https://www.lovd.nl/), and was, therefore, submitted to the gene‐specific LOVD database (https://databases.lovd.nl/shared/variants/LTBP4/; DB‐ID: LTBP4_000036).

## 4 DISCUSSION

Our report highlights the importance of clinical expertise to address targeted molecular analysis, which, in turn, allows a definite diagnosis, in patients suggestive of ARCL1C, also considering the absence of formal diagnostic criteria due to the limited number of reported patients. Indeed, until now only 18 patients (Callewaert et al., 2013; Su et al., 2015; Urban et al., 2009; this work) from 14 families are described (Table 1). Cutis laxa and dysmorphism were evident from birth in all patients (18/18). The most frequently observed craniofacial features were depressed nasal bridge with anteverted nares (9/10), narrow forehead (9/11), hypertelorism (7/9), periorbital swelling (6/9), and long philtrum (9/12). Consistently, our patient presented severe cutis laxa, mainly localized to face, thorax, and abdomen, resulting in a coarse/aged appearance, and all the abovementioned dysmorphism. Severe pulmonary emphysema was present in all patients (13/13), except ours, and represented the most common cause of death (10/12). Indeed, the overall prognosis is poor, with a mortality rate of 72% (13/18). Mean age at death was 2.4 years (median age 6 months). The five surviving patients were all female (ages 1.5–23 years). In addition to pulmonary emphysema, brain abscess and gastric perforation were each reported once as a cause of death.

**Table 1:** Summary of clinical features of all patients with *LTBP4* pathogenic variants

Citations	Our Patient	Urban et al. (2009)				Callewaert et al. (2013)									Su et al. (2015)			
	P1	P2	P3	P4	P5	P6	P7	P8	P9	P10	P11	P12	P13	P14	P15	P16	P17	P18
Sex	F	M	M	F	F	F	M	F	M	M	M	F	F	M	M	F	F	M
Age	18 month	9 month	4 month	7 year	26 month	23 years	4 weeks	3 months	2 years	10 years	6 months	6 months	13 years	6 weeks	15 months	14 years	20 years	6 weeks
Cause of death	−	PE	PE	−	PE	‐	PE	PE	PE	PE	PE PHrT	PHrT GP	BA	PE	NA	−	−	BD
Skin																		
Cutis laxa	+	+	+	+	+	+	+	+	+	+	+	+	+	+	+	+	+	+
Craniofacial																		
Long philtrum	+	+	+	+	NA	−	NA	NA	+	NA	NA	+	+	−	−	+	+	NA
Fat midface	+	+	+	+	NA	−	NA	NA	−	NA	NA	−	−	−	−	NA	NA	NA
Narrow forehead	+	+	+	NA	+	−	NA	NA	+	NA	NA	+	+	+	−	+	NA	NA
Periorbital swelling	+	+	+	NA	NA	−	NA	NA	+	NA	NA	+	−	−	+	NA	NA	NA
Hypertelorism	+	+	+	NA	NA	−	NA	NA	+	NA	NA	+	+	−	+	NA	NA	NA
Depressed nasal bridge anteverted nares	+	+	+	NA	NA	+	NA	NA	+	NA	NA	+	−	+	+	+	NA	NA
Retrognathia, micrognathia or mandibular hypoplasia	+	+	+	+	+	−	NA	NA	−	NA	NA	−	−	−	−	NA	NA	NA
Wide suture or fontanels	+	+	+	NA	−	−	−	−	−	−	−	−	−	+	−	NA	NA	NA
Pulmonary																		
Tachypnea, respiratory distress	+	+	+	+	+	+	+	+	+	+	+	+	+	+	+	+	NA	NA
Pneumonia	+	+	−	−	+	NA	NA	NA	NA	NA	NA	NA	NA	NA	+	+	NA	+
Laryngomalacia Tracheomalacia Bronchomalacia	−	−	+	NA	+	−	+	−	−	−	−	−	−	+	−	NA	NA	NA
Diaphragmatic hernia or eventration	+	−	+	+	+	+	−	−	+	+	−	+	−	+	+	NA	NA	NA
Emphysema	−	+	+	NA	+	+	+	+	+	+	+	+	+	+	+	NA	NA	NA
Gastrointestinal																		
Diverticula	−	−	+	+	NA	−	−	−	−	−	−	+	−	−	+	NA	NA	+
Intestinal dilation, tortuosity	+	+	+	NA	−	−	−	−	−	−	+	−	−	−	+	NA	NA	NA
Umbilical hernia	+	+	−	−	+	NA	NA	NA	NA	NA	NA	NA	NA	NA	NA	NA	NA	NA
Gastrointestinal reflux	−	−	−	+	+	NA	NA	NA	NA	NA	NA	NA	NA	NA	NA	NA	NA	NA
Rectal prolapse	−	−	−	+	+	−	−	−	−	+	−	−	−	−	NA	NA	NA	NA
Pyloric stenosis	−	−	+	−	+	NA	NA	NA	NA	NA	NA	NA	NA	NA	NA	NA	NA	NA
Genitourinary																		
Bladder diverticula	−	+	+	+	NA	+	−	+	−	+	−	−	+	+	+	NA	−	+
Hydronephrosis	+	+	+	−	NA	−	+	−	−	−	−	−	−	+	NA	NA	NA	NA
Inguinal hernia	−	+	+	−	−	−	−	−	−	+	−	−	−	−	+	NA	NA	NA
Cardiovascular																		
Peripheral pulmonary artery stenosis	−	+	−	NA	+	+	+	−	+	−	−	+	+	−	+	+	+	+
Atrial septal defect or aneurysms	+	−	−	NA	−	−	−	−	−	+	−	+	−	−	−	−	+	+
Cardiac valve insufficiency	−	−	−	NA	−	−	−	−		+	−	+	−	+	+	+	−	−
Pulmonary or aortic valve stenosis	−	−	−	NA	+	−	−	−	−	−	−	−	−	−	+	−	−	−
Pulmonary hypertension	−	+	−	NA	+	−	−	−	−	−	+	+	+	+	+	−	+	−
Patent foramen ovale	−	+	+	−	NA	−	−	−	−	−	−	−	−	+	+	−	−	−
Other																		
Joint laxity	+	+	+	+	+	+	−	−	−	−	−	−	−	+	NA	+	+	+
Muscular hypotonia	+	+	+	NA	+	−	+	−	+	−	−	+	−	+	+	NA	NA	+
Foot deformity	−	+	+	NA	+	NA	NA	NA	NA	NA	NA	NA	NA	NA	NA	+	+	NA

Abbreviations: BD, breathing difficulties; Ba, brain abscesses; GP, gastric perforation; PE, pulmonary emphysema; PHrT, pulmonary hypertension; NA, not available.

Diaphragmatic hernia or eventration was also common (10/15) as well as bladder (10/16) and gastrointestinal diverticula (5/15), intestinal dilatation/tortuosity (5/14), and hydronephrosis (5/13). Up to the moment of evaluation, our patient only presented episodes of respiratory distress, diaphragmatic eventration, intestinal dilation and tortuosity, and hydronephrosis. However, multidisciplinary evaluations are planned including immunizations against respiratory infections and periodic assessment of pulmonary function and imaging of gastrointestinal and urinary tracts. The absence of pulmonary emphysema in our patient is noteworthy but it is not easy to explain. In our opinion, it is most likely due to clinical variability rather than to the specific type of pathogenic variant or the young age of the patient. Indeed, the patient's variant should represent a null allele, as the majority of the mutations reported so far (Table 2), and fatal pulmonary emphysema was described in as many as eight patients who were younger than ours (Table 1).

**Table 2:** *LTBP4* pathogenic variants

Patient	Cons.	Status	Exon	cDNA	Protein	Type	Domains	References
P1	+	Homozygous	12	c.1450del	p.(Arg484Glyfs*290)	Frameshift‐PTC	‐	This report
P2	+	Homozygous	28	c.3554del	p.(Gln1185Argfs*27)	Frameshift‐PTC	Second 8‐Cys domain	Urban et al. (2009)
P3	−	Compound Heterozygous	9	c.791del	p.(Pro264Argfs*37)	Frameshift‐PTC	Hybrid domain	
			22	c.2570_2571delGCinsAA	p.(Cys857*)	Frameshift‐PTC	Eleventh EGF‐like domain	
P4	+	Homozygous	9	c.820T>G	p.(Cys274Gly)	Missense	Hybrid domain	
P5	−	Compound Heterozygous	22	c.2570_2571delGCinsAA	p.(Cys857*)	Frameshift‐PTC	Eleventh EGF‐like domain	
			33	c.4127dup	p.(Arg1377Alafs*27)	Frameshift‐PTC	Third 8‐Cys domain	
P6	−	Compound Heterozygous	11	c.1342C>T	p.(Arg448*)	Nonsense	First 8‐Cys domain	Callewaert et al. (2013)
			31	c.4115dup	p.(Tyr1373Ilefs*2)	Frameshift‐PTC	Third 8‐Cys domain	
P7	+	Homozygous	19	c.2408C>A	p.(Ser803*)	Nonsense	Seventh EGF‐like domain	
P8	−	Compound Heterozygous	28	c.3661C>T	p.(Gln1221*)	Nonsense	Second 8‐Cys domain	
			29	c.3886C>T	p.(Gln1296*)	Nonsense	Fourteenth EGF‐like domain	
P9		Homozygous	6	c.780+2T>G	‐	Splicing	‐	
P10	+	Homozygous	11	c.1263del	p.(Cys422Alafs*352)	Frameshift‐PTC	First 8‐Cys domain	
P11	+	Homozygous	15	c.1851C>A	p.(Cys617*)	Nonsense	Second EGF‐like domain	
P12	+	Homozygous	31	c.4127dup	p.(Arg1377Alafs*27)	Frameshift‐PTC	Third 8‐Cys domain	
P13	+	Homozygous	31	c.4128C>T	p.(Arg1377*)	Nonsense	Third 8‐Cys domain	
P14	+	Homozygous	26	c.3556T>C	p.(Cys1186Arg)	Missense	Second 8‐Cys domain	
P15	−	Compound Heterozygous	7	c.883+1G>T	‐	Splicing	Hybrid domain	Su et al. (2015)
			17	c.2161C>T	p.(Arg721*)	Nonsense	Eighth EGF‐like domain	
P16	−	Compound Heterozygous	18	c.2377_2378insA	p.(Gly793Glufs*5)	Frameshift‐PTC	Ninth EGF‐like domain	
			29	c.3856T>A	p.(Cys1286Ser)	Missense	Eighteenth EGF‐like domain	
P17	−	Compound Heterozygous	20	c.2632G>T	p.(Gly878*)	Nonsense	Eleventh EGF‐like domain	
			31	c.4113dup	p.(Ala1372Argfs*3)	Frameshift‐PTC	Third 8‐Cys domain	
P18	+	Homozygous	5	c.341−1G>C	‐	Splicing	First EGF‐like domain	

Exons and mutations numbering are based on transcript NM_003573.2, NP_003564.2; Cons, consanguinity; PTC, premature termination codon.

Concerning the cardiovascular phenotype, peripheral pulmonary artery stenosis (11/17) and pulmonary hypertension (8/17) are common features in addition to arterial septal defect (5/17) and cardiac valve insufficiency (5/17). Other prevalent findings are muscular hypotonia (10/15) and joint hypermobility, usually of small joints (10/17) (Table 1).

*LTBP4* encodes a member of the latent transforming growth factor‐beta (TGFβ) binding proteins (LTBPs) that are structurally related to fibrillins. LTBP4 binds the small latent complex (SLC) consisting of TGFβ1 and its latency‐associated peptide. This interaction allows LTBP4 to sequester TGFβ1 and control its activation. LTBP4 also enhances elastogenesis by regulating the incorporation of elastin‐fibulin‐5 complexes into the microfibrillar bundles to form elastic fibers (Callewaert et al., 2013). In addition, LTBP4 stabilizes the TGFβ receptors and loss of LTBP4 results in diminished TGFβ signaling (Callewaert & Urban, 2016; Su et al., 2015). The majority (19/23) of the currently described pathogenic variants (Table 2) is frameshift (8/23), nonsense (8/23), and splice variants (3/23) resulting in a PTC and activation of the nonsense‐mediated mRNA decay (NMD), as demonstrated by qPCR analysis (Callewaert et al., 2013). In the absence of LBTP4 protein, fibulin‐5‐elastin complexes fail to target the microfibrils, resulting in severely impaired elastic fiber formation and altered TGFβ signaling (Callewaert et al., 2013; Dabovic et al., 2015; Urban et al., 2009). One exception, the recurrent c.4127dup variant, was described to result in a C‐terminal truncated LTPB4 protein (p.Arg1377Alafs*27) with a presumed gain‐of‐function mechanism (Callewaert et al., 2013). Furthermore, few missense substitutions (3/23) are reported, which cause the loss of one of the highly conserved cysteine residues located in a TGFβ‐binding (TB) domain or hybrid domain that are implicated in binding of the SLC (Table 2). Loss of these cysteine residues was shown to interfere with the conformation and function both in LTBPs and fibrillin (Jensen, Iqbal, Lowe, Redfield, & Handford, 2009; Lack et al., 2003). The novel pathogenic frameshift variant c.1450del (p.Arg484Glyfs*290) identified in the present study is predicted, with a high degree of confidence, to activate the NMD; however, the real functional outcome was not investigated, since patient's fibroblasts were not available.

## 5 CONCLUSIONS

Our findings expand both the knowledge of the clinical phenotype and the allelic repertoire of ARCL1C. Further reports are needed to better characterize the *LTBP4*‐related phenotype and define specific clinical criteria that might facilitate the differential with other ARCL1 subtypes, delineate genotype–phenotype correlations, and collect natural history data for prognostication.

## CONFLICT OF INTEREST

The authors declare no conflict of interest.

## ETHICAL APPROVAL

The patient's parents provided written informed consent for genetic testing and publication of clinical data and photographs. This study follows the Helsinki Declaration's principles and was carried out from routine diagnostic activity; formal ethics review was therefore not requested.
